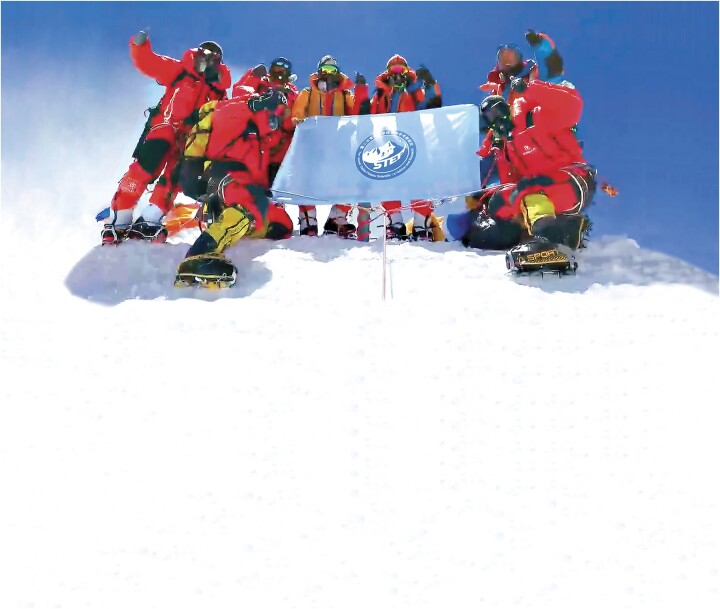# China's TOP10 breakthroughs in science and technology in 2022

**DOI:** 10.1093/nsr/nwad058

**Published:** 2023-03-06

**Authors:** He Zhu

**Affiliations:** science and news editor at the editorial office of NSR in Beijing, China

## Abstract

The members of Chinese Academy of Sciences (CAS) and Chinese Academy of Engineering have, for 29 years, selected every year's top 10 advancements in science and technology by domestic researchers. For 2022, the list was announced on 12 January, 2023 in *China Science Daily*. This year's collection includes 4 entries in space exploration and observation, 2 in biotechnology related to agriculture, 2 in earth and environmental sciences, and 2 in fundamental physics.

1. Discoveries by the Five-hundred-meter Aperture Spherical radio Telescope (FAST)

FAST is a radio dish located in Guizhou Province that detects radio signals from outer space. With its 500 meter diameter, it is the world's largest and most sensitive radio telescope of its kind. The chief scientist at FAST, Prof. Di Li and his team at the National Astronomical Observatories (NAO), in collaboration with scientists around the world, has made a series of discoveries in astronomy throughout 2022: polarization measurements of five repeating fast radio bursts (FRBs), providing crucial evidence of FRBs’ origins [1]; discovery of a repeating fast radio burst FRB 20190520B, using its dispersion and scattering to assess its host galaxy's plasma properties. Another international team led by NAO and Peking University measured dynamic behaviors of magnetic fields within 1 astronomical unit of the source of FRB 20201124A. In addition, Prof. Cong Xu's team at NAO studied atomic gas contents of four low-redshift galaxies using FAST.

**Figure 1.**
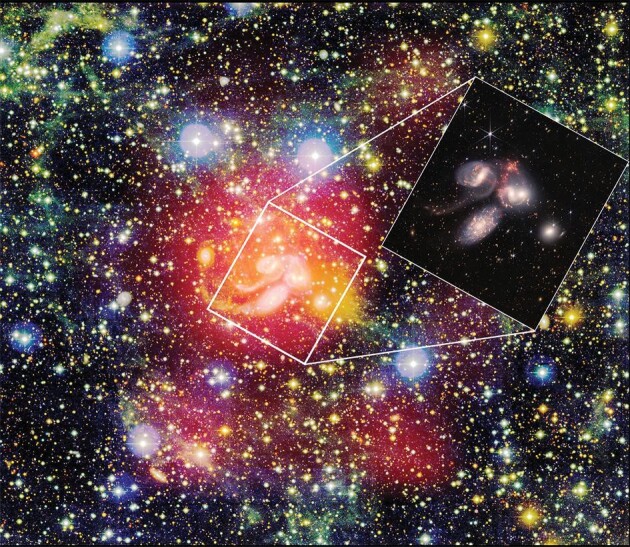
FAST observations of atomic hydrogen in the vicinity of Stephen's Quintet.

2. Construction completed at China Space Station

On 29 November, 2022, Longmarch-2 rocket carried Shenzhou-15 spacecraft to the orbit and completed docking with China Space Station (CSS) on the 30^th^. CSS now consists of Tianhe core module and two experiment modules, Wentian and Mengtian, forming a T-shaped structure. Shenzhou-15 joined Shenzhou-14 spacecraft and Tianzhou-5 cargo ship. Together, the triple-module and triple-ship layout had reached its maximal designed capacity with a total mass close to 100 tons. The crew of Shenzhou-15 moved in CSS and celebrated the first ‘Space Family’ from China with the crew of Shenzhou-14. Shenzhou-14 safely returned on 4 December, concluding a 183-day mission. This changeover marks the beginning of continuous manned mission at CSS.

**Figure 2.**
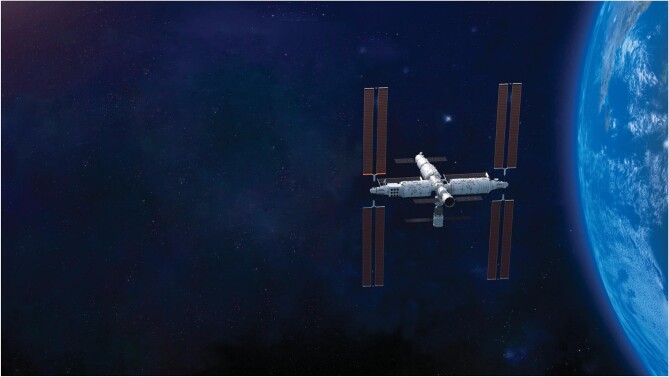
The maximal designed capacity of China Space Station: triple-module and triple-ship.

3. Discoveries of common genes in corn and rice to improve yields

A research team led by Profs. Jiansheng Li and Xiaohong Yang from China Agriculture University and Prof. Jianbing Yan from Huazhong Agriculture University discovered that KRN2 gene in corn and OsKRN2 gene in rice experienced convergent selection, a phenomenon among genes of different species that share similar functions and ancestry [2]. Their common function, in this case, entails encoding WD40 proteins to negatively regulate grain number. Consequently, perturbing these genes increased grain yield by 10% in corn and 8% in rice, respectively. Genome-wide analysis is underway to better understand their lineage in evolution. This discovery not only reveals a mechanism of increasing productions of corn and rice, but also provides theoretical foundation for enhancements of other plants.

**Figure 3.**
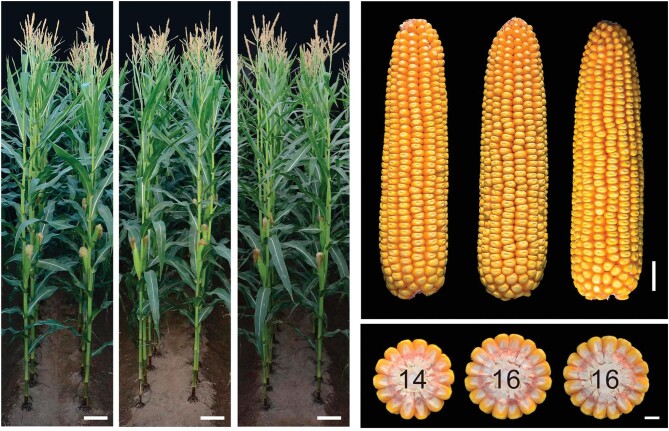
Genetic modification of corn results in 10% increase in grain yield.

4. Discovery of metallic behaviors in a bosonic high-temperature superconductor

Prof. Yanrong Li of University of Electronic Science and Technology of China, Prof. Xincheng Xie of Peking University and Prof. James M. Valles Jr. of Brown University together demonstrated strange metallic behaviors in a bosonic system, a high-temperature superconductor (YBa2Cu3O7) [3]. Fundamental particles are divided into fermions and bosons based on their statistical properties. Almost all materials behind electronic devices in our daily lives are systems of fermions. Their resistivity is explained through the formation of quasi-particles. Our understanding of their electrical properties implies the path of shrinking devices is near the end due to power consumption. Bosonic systems, such as some high-temperature superconductors, do not appear to be governed by the quasi-particle principle and possess superior energy efficiency. This discovery greatly enhances our knowledge of bosonic systems of condensed matter and paves the way for future quantum computation technology and high sensitivity quantum detection.

**Figure 4.**
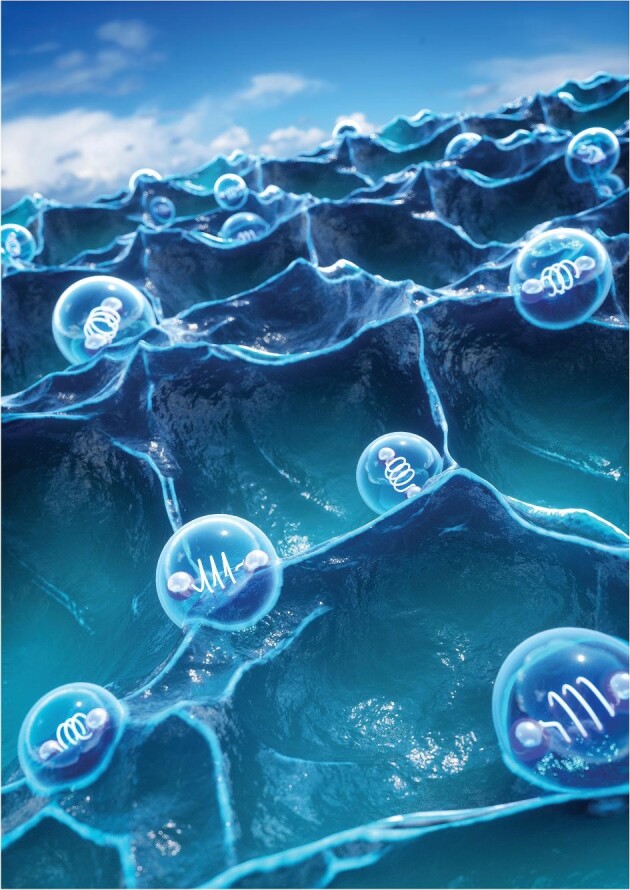
Strange metallic behaviors discovered in a bosonic system: nanopatterned YBa2Cu3O7-delta (YBCO) film arrays.

5. Artificial synthesis of glucose and fatty acids from CO_2_

Chinese scientists at Tianjin Institute of Industrial Biotechnology, CAS made the breakthrough discovery of artificial synthesis of starch using CO_2_ in a cell-free system. This achievement may generate broad impacts in terms of the conservation of water resources and land use around the world. In 2022, a cross-country team consisting of experts in University of Electronic Science and Technology of China (Chengdu), University of Science and Technology of China (Hefei) and Shenzhen Institute of Advanced Technology, CAS (Shenzhen) pushed the frontier of this area even further by achieving artificial synthesis of glucose and fatty acids from CO_2_ [4]. This innovation features a hybrid electro-biosystem by incorporating a nanostructured copper catalyst into CO_2_ electrolysis with yeast fermentation. Along with starch synthesis, these processes highlight a tantalizing future of renewable-agriculture running on electricity.

**Figure 5.**
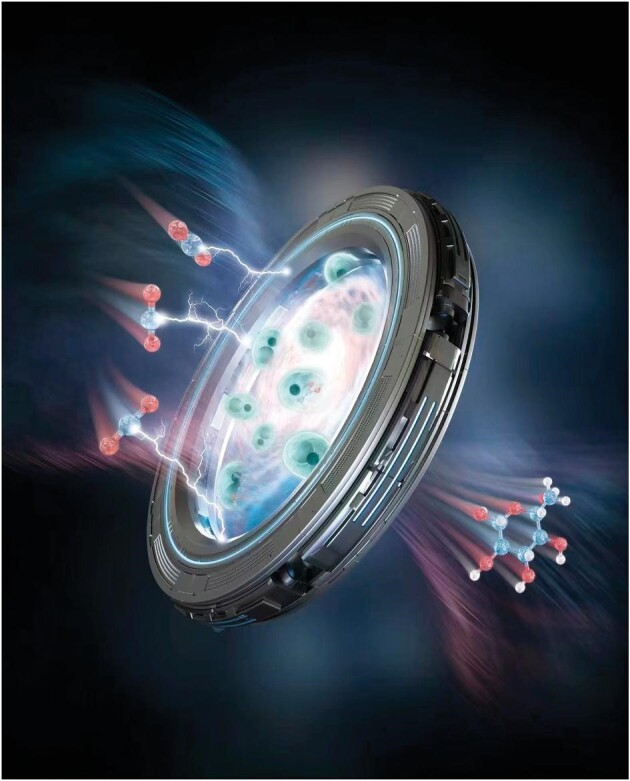
CO_2_ electrolysis using a hybrid electro-biosystem with yeast fermentation.

6. Successful debut of China's most powerful solid-propellant carrier rocket: Lijian-1 (ZK-1A)

The historic launch took place at Jiuquan Satellite Launch Center at 12 : 12 on 27 July. The Institute of Mechanics, CAS led the design effort and achieved the original goal of six satellites on one rocket. As a preferred launch platform of small to mid-sized satellites, the Lijian-1 now enhances the over-all launch capacity of China's solid-propellant rockets. The four-stage rocket measures 30 meters in length and 2.65 meters in diameter. The designed launch weight is 135 tons with a maximal thrust of 200 tons. The maximal payload for a 500-kilometer heliosynchronous orbit is 1500 kilograms. The Lijian-1 project was funded as a key component of the 14^th^ five-year plan of CAS to strengthen China's space science and technology.

**Figure 6.**
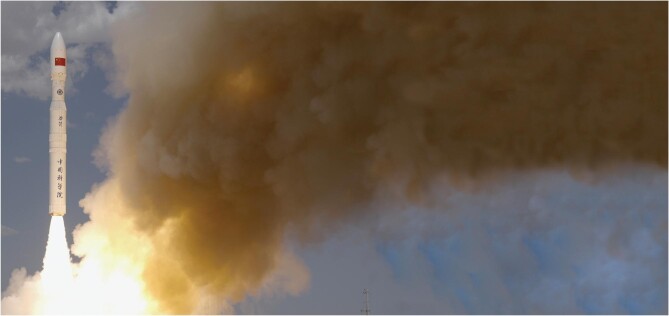
Successful debut of China's most powerful solid-propellant carrier rocket: Lijian-1 (ZK-1A)

7. Successful launch of the Kuafu-1 satellite: Advanced Space-based Solar Observatory (ASO-S)

The solar images acquired by Kuafu-1, first made public on 13 December, were collected by three instruments in two months: a coronagraph measuring the corona within and beyond the visible light spectrum, a magnetograph measuring the sun's magnetic field and an X-ray camera for imaging of high energy bursts in solar flares. As a comprehensive science satellite dedicated for solar detection, ASO-S was co-developed by a national consortium of CAS institutions [5]. The system design and ground support were developed by the National Space Science Center. The satellite and its onboard instruments were designed and manufactured by Innovation Academy for Microsatellites, National Astronomical Observatories, Changchun Institute of Optics, Fine Mechanics and Physics and the Purple Mountain Observatory. The satellite was monitored and operated by China Satellite Monitoring and Control Center and the launch rocket was manufactured by China Aerospace Science and Technology Corporation.

**Figure 7.**
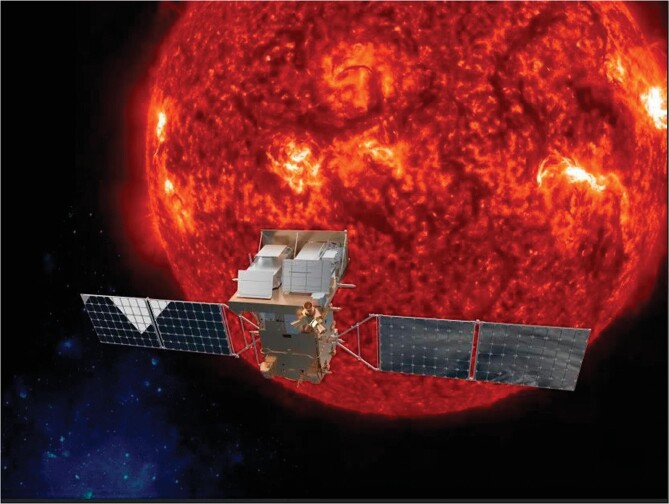
Kuafu-1’s missions include corona measurements and imaging of high energy bursts in solar flares.

8. Hydrogen generation technology to electrolyze seawater without desalination

The shortage of fresh water worldwide requires us to look tothe ocean as water supply of hydrogen-based energy in the future. However, due the complex composition of seawater, electrolyzing seawater remains a great challenge and pre-desalination before electrolysis is not cost effective. Prof. Heping Xie, a member of the Chinese Academy of Engineering, led teams at Shenzhen University and Sichuan University and developed a new method to directly electrolyze seawater based on new electrochemical principles [6]. This technology enables hydrogen generation without side-reactions and corrosion problems and it does not require desalination. Combined with ocean-based renewable energy sources such as wind or tidal energy, this concept may create a complete ocean-side energy generation and storage system that only outputs hydrogen without pollution.

**Figure 8.**
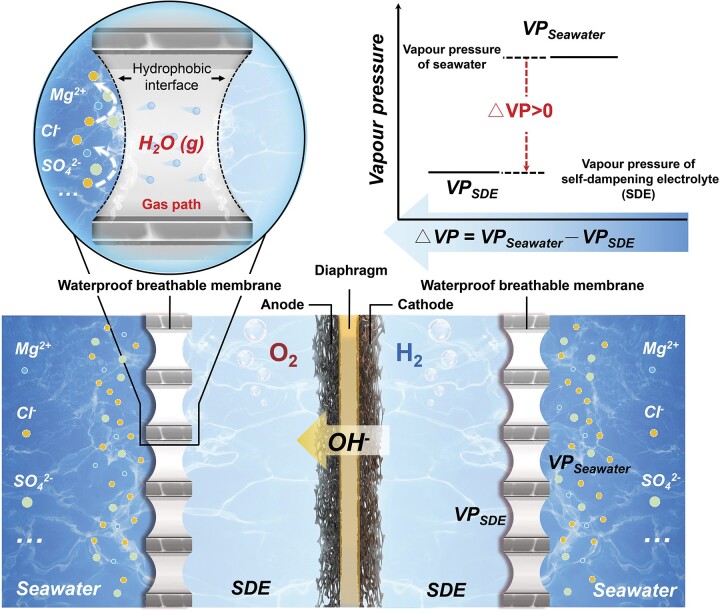
Scientific principles of seawater electrolysis.

9. World record of highest magnetic field broken by China's Steady High Magnetic Field Facility

As part of the major national science and technology infrastructure, China's Steady High Magnetic Field Facility in Hefei broke the world record of the highest magnetic field on 12 August. The 23-year old record of 45 Tesla was replaced by the new value of 45.22 Tesla. This facility is operated by the High Magnetic Field laboratory at Hefei Institutes of Physical Science, CAS. It was initiated as part of the 11^th^ five-year plan for large scale facilities of basic sciences. A steady high magnetic field is an extreme experimental condition needed in many physical sciences to enable break-through discoveries. An extremely high magnetic field may induce exotic changes in materials’ properties and may reveal new phenomena and new principles. Setting this new record is a major milestone in the development of extreme experimental conditions in China.

**Figure 9.**
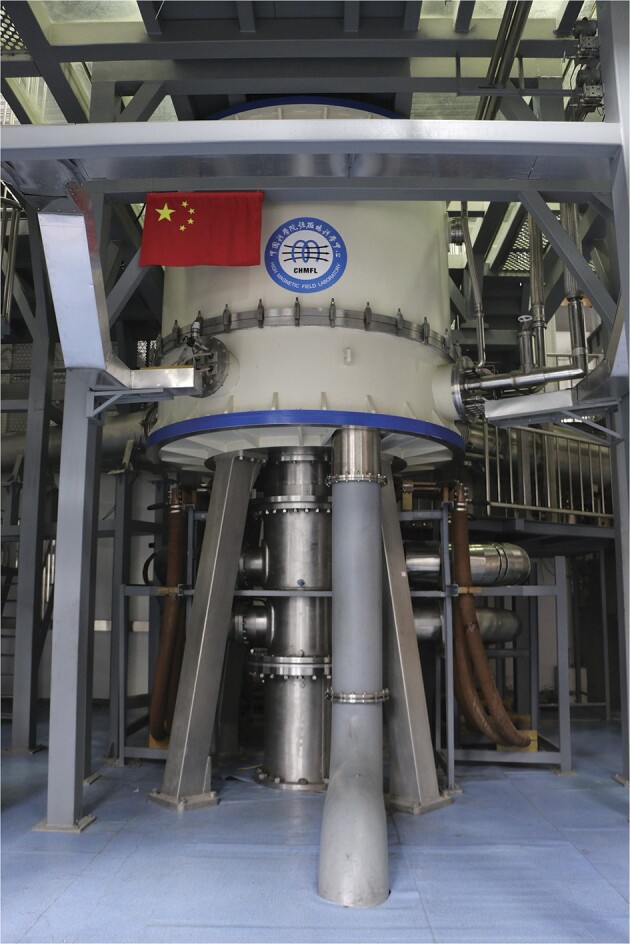

10. Earth Summit Mission set multiple records at Mount Qomolangma

As the highlight of the Second Tibetan Plateau Scientific Expedition and Research (STEP), the Earth Summit Mission was completed on 30 May by a Chinese expedition team at Mount Qomolangma. The primary objectives of this mission include detecting the vertical structure of weather parameters such as wind, temperature, humidity, and pressure with advanced instruments. The mission successfully established the first vertical network of weather detectors and performed real-time data transfer. The team also constructed the highest weather station in the world at an altitude of 8830 meters, as a part of the vertical network starting from 4276 meters. In addition, the expedition team performed measurements using an aerostat (Jimu-1) that measures 55 meters in length and 19 meters in height with a volume of 9060 cubic meters. This vehicle set the world record of highest weather measurement of its kind at 9050 meters in altitude. Other interesting scientific measurements include snow thickness measured by high precision radar and ozone density measurement at 39 kilometers from surface.

**Figure 10.**